# Silencing salusin-β attenuates cardiovascular remodeling and hypertension in spontaneously hypertensive rats

**DOI:** 10.1038/srep43259

**Published:** 2017-02-23

**Authors:** Xing-Sheng Ren, Li Ling, Bing Zhou, Ying Han, Ye-Bo Zhou, Qi Chen, Yue-Hua Li, Yu-Ming Kang, Guo-Qing Zhu

**Affiliations:** 1 Key Laboratory of Cardiovascular Disease and Molecular Intervention, Department of Physiology, Nanjing Medical University, Nanjing, Jiangsu 211166, China; 2 Department of Pathophysiology, Nanjing Medical University, Nanjing, Jiangsu 211166, China; 3 Department of Physiology and Pathophysiology, Cardiovascular Research Center, Xi’an Jiaotong University School of Medicine, Xi’an 710061, China

## Abstract

Salusin-β is a bioactive peptide involved in vascular smooth muscle cell proliferation, vascular fibrosis and hypertension. The present study was designed to determine the effects of silencing salusin-β on hypertension and cardiovascular remodeling in spontaneously hypertensive rats (SHR). Thirteen-week-old male SHR and normotensive Wistar-Kyoto rats (WKY) were subjected to intravenous injection of PBS, adenoviral vectors encoding salusin-β shRNA (Ad-Sal-shRNA) or a scramble shRNA. Salusin-β levels in plasma, myocardium and mesenteric artery were increased in SHR. Silencing salusin-β had no significant effect on blood pressure in WKY, but reduced blood pressure in SHR. It reduced the ratio of left ventricle weight to body weight, cross-sectional areas of cardiocytes and perivascular fibrosis, and decreased the media thickness and the media/lumen ratio of arteries in SHR. Silencing salusin-β almost normalized plasma norepinephrine and angiotensin II levels in SHR. It prevented the upregulation of angiotensin II and AT_1_ receptors, and reduced the NAD(P)H oxidase activity and superoxide anion levels in myocardium and mesenteric artery of SHR. Knockdown of salusin-β attenuated cell proliferation and fibrosis in vascular smooth muscle cells from SHR. These results indicate that silencing salusin-β attenuates hypertension and cardiovascular remodeling in SHR.

Salusin-β is identified to be a bioactive peptide of 20 amino acids with mitogenic effect in 2003, which is translated from an alternatively spliced mRNA of torsion dystonia-related gene (TOR2A)^1^. The initial 18 amino acids of human salusin-β have high homology with the N-terminal sequence of rat salusin^2^. Salusin-β is widely expressed in central and peripheral tissues^2^,^3^. Plasma salusin-β levels were distinctly increased in subjects with diabetes mellitus, coronary artery disease, and cerebrovascular disease compared with healthy controls, and it may be an indicator of systemic vascular diseases^4^. Salusin-β is involved in hypertension^5^. We have found that central salusin-β is involved in sympathetic activation, arginine vasopressin release and hypertension^6^,^7^,^8^ and plasma salusin-β level was increased in renovascular hypertensive rats^7^. Central blockade of salusin β attenuates hypertension^9^. Recently, we have showed that intravenous injection of salusin-β dose-dependently increases blood pressure, but excessive salusin-β reduces blood pressure due to its bradycardia effect^10^. Salusin-β overexpression causes severe hypertension in rats.

Hypertension is involved in large and small vascular remodeling that impacts cardiovascular prognosis^11^. Indices of small resistance artery structure, such as the ratio of media to internal lumen, may have a strong prognostic significance in hypertensive patients^12^. The structure of arteries is dependent not only on blood pressure but also on several other factors including blood flow and hormonal environment^13^. Hypertension partially contributes to vascular remodeling, which reinforce the development of hypertension, thus reflecting a vicious circle^14^. Left ventricular hypertrophy and remodeling are frequently seen in hypertensive subjects and consistently associated with increased cardiovascular morbidity and mortality^15^. We have found that salusin-β induces foam cell formation and monocyte adhesion in human vascular smooth muscle cells (VSMCs)^16^. Salusin-β promotes VSMCs migration and intimal hyperplasia after vascular injury^17^. It stimulates human VSMCs proliferation via cAMP-PKA-EGFR-CREB/ERK pathway, and causes vascular fibrosis via TGF-β1-Smad pathway^10^. However, it is unknown whether endogenous salusin-β plays a role in the pathogenesis of hypertension and cardiovascular remodeling. Spontaneously hypertensive rats (SHR) is a commonly used animal model of primary hypertension. The genetic hypertension model provides many similarities to human essential hypertension in pathophysiological development, neuroendocrine changes, clinical courses and secondary diseases^18^,^19^. Thus, SHR was used as a hypertension animal model in the present study. The aim of this study is to determine whether endogenous salusin-β contributes to hypertension and cardiovascular remodeling.

## Results

### Salusin-β expression

**Figure 1:**
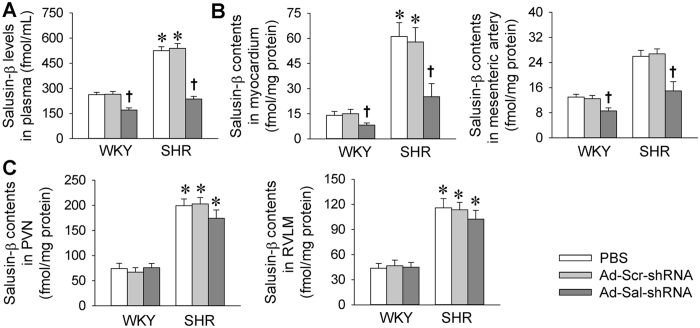
Salusin-β levels in WKY and SHR. The measurements were carried out 2 weeks after intravenous injection of PBS, adenoviral vectors encoding scramble shRNA (Ad-Scr-shRNA) or salusin-β shRNA (Ad-Sal-shRNA). (**A**) Plasma salusin-β levels. (**B**) Salusin-β contents in myocardium and mesenteric artery. (**C**) Salusin-β contents in hypothalamic paraventricular nucleus (PVN) and rostral ventrolateral medulla (RVLM) of the brain. Values are mean ± S.E.M. ^*^P < 0.05 vs. WKY. ^†^P < 0.05 vs. PBS or Ad-Scr-shRNA. n = 6 for each group.

Plasma salusin-β levels were increased more than twofold in SHR compared with WKY. Silencing salusin-β with intravenous administration of adenoviral vectors encoding salusin-β shRNA (Ad-Sal-shRNA) reduced the plasma salusin-β levels in both WKY and SHR (Fig. 1A). Similarly, salusin-β contents in myocardium and mesenteric artery were increased about fourfold and twofold, respectively, compared with WKY. Ad-Sal-shRNA reduced the salusin-β contents of myocardium and mesenteric artery in both WKY and SHR (Fig. 1B). Although the salusin-β contents in the hypothalamic paraventricular nucleus (PVN) and rostral ventrolateral medulla (RVLM) of the brain was increased in SHR, intravenous administration of Ad-Sal-shRNA had no significant effect on the salusin-β contents in the PVN and RVLM (Fig. 1C).

### Blood pressure and heart rate

**Figure 2:**
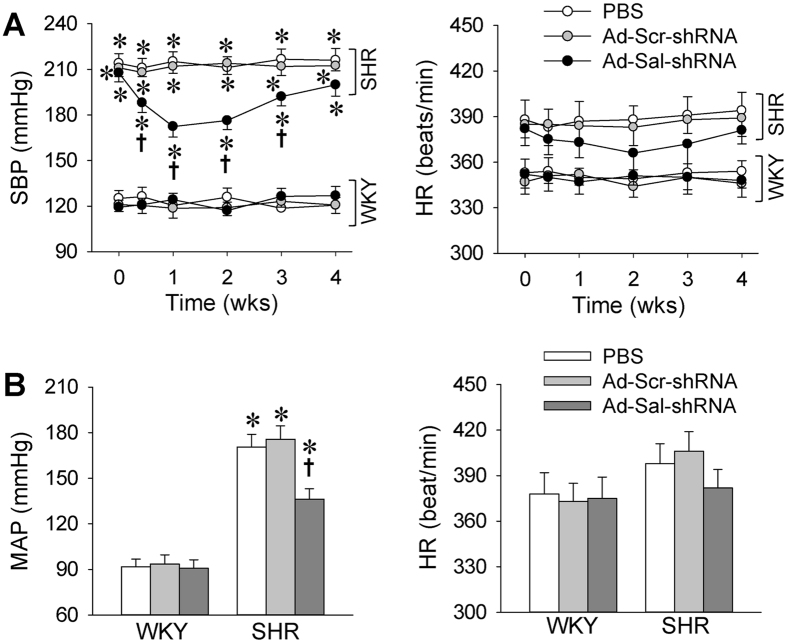
Arterial blood pressure and heart rate in WKY and SHR. (**A**) Systolic blood pressure (SBP) and heart rate (HR) in conscious state before and after intravenous injection of PBS, adenoviral vectors encoding scramble shRNA (Ad-Scr-shRNA) or salusin-β shRNA (Ad-Sal-shRNA). (**B**) Mean arterial pressure (MAP) and HR in anesthetized state 2 weeks after the injections. Values are mean ± S.E.M. ^*^P < 0.05 vs. WKY. ^†^P < 0.05 vs. PBS or Ad-Scr-shRNA. n = 6 for each group.

Systolic blood pressure (SBP) of tail artery measured in the conscious state in SHR was much higher than that in WKY. Adenoviral vectors encoding salusin-β shRNA (Ad-Sal-shRNA) had no significant effect on SBP in WKY, but caused a significant decrease in SBP in SHR from the 1st week to the 3rd week day after the Ad-Sal-shRNA administration. However, Ad-Sal-shRNA had no significant effect on heart rate (HR) in both WKY and SHR. The maximal depressor effect in SHR (SBP: −35.2±4.4 mmHg, *P* < 0.001) was observed at the 1st week after administration of Ad-Sal-shRNA (Fig. 2A). Therefore, all the acute experiments were carried out at the end of the 2nd week after the intravenous intervention in the following experiments. To confirm the depressor effect of Ad-Sal-shRNA, mean arterial pressure (MAP) and HR were measured during acute experiment in anesthetized state. MAP in anesthetized state in SHR was much higher than that in WKY. Ad-Sal-shRNA caused a significant decrease in MAP in SHR, but had no significant effect on the MAP in WKY as well as the HR in both WKY and SHR (Fig. 2B).

### Left ventricular hypertrophy and remodeling

**Figure 3:**
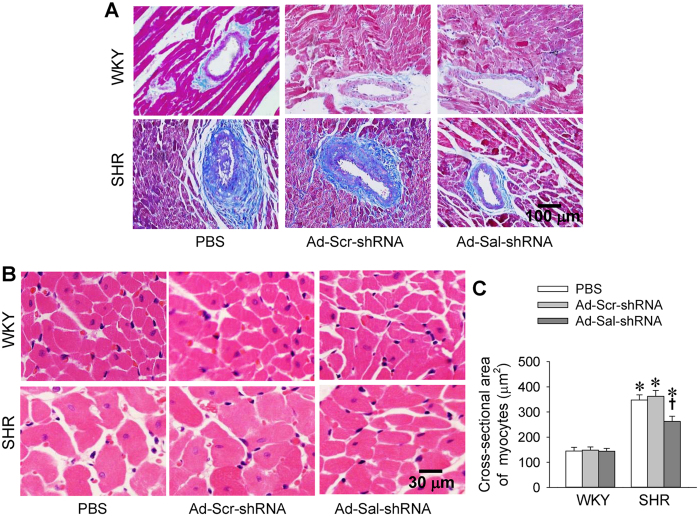
Perivascular fibrosis in myocardium and cross-sectional area of cardiomyocytes in WKY and SHR. The measurements were carried out 2 weeks after intravenous injection of PBS, adenoviral vectors encoding scramble shRNA (Ad-Scr-shRNA) or salusin-β shRNA (Ad-Sal-shRNA). (**A**) Sections with Masson’s stain showing perivascular fibrosis in myocardium. (**B**) Sections with HE stain showing the size of cardiomyocytes. (**C**) Bar graph showing quantitative analysis of cross-sectional area of cardiomyocytes. Values are mean ± S.E.M. ^*^P < 0.05 vs. WKY. ^†^P < 0.05 vs. PBS or Ad-Scr-shRNA. n = 6 for each group.

Left ventricular weight (LVW) and the ratio of LVW to body weight (BW) were increased in SHR, which were reduced by Ad-Sal-shRNA (Table 1). Slight fibrosis in myocardium and severe perivascular fibrosis were observed in SHR, which were attenuated by Ad-Sal-shRNA (Fig. 3A). Cardiomyocyte hypertrophy and increased cross-sectional area of cardiomyocytes were found in SHR, The cross-sectional area of cardiomyocytes was significantly increased in SHR, which was blunted by Ad-Sal-shRNA (Fig. 3B,C).

### Vascular remodeling

**Figure 4:**
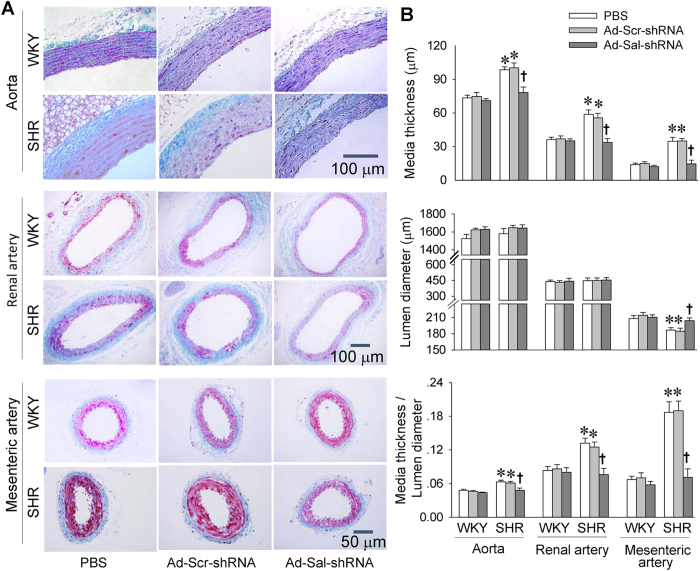
Vascular remodeling of aorta, main renal artery and mesenteric artery in WKY and SHR. The measurements were carried out 2 weeks after intravenous injection of PBS, adenoviral vectors encoding scramble shRNA (Ad-Scr-shRNA) or salusin-β shRNA (Ad-Sal-shRNA). (**A**) Representative transverse section images with Masson’s stain. (**B**) Bar graph showing the media thickness, lumen diameter and the ratio of media thickness to lumen diameter. Values are mean ± S.E.M. ^*^P < 0.05 vs. WKY. ^†^P < 0.05 vs. PBS or Ad-Scr-shRNA. n = 6 for each group.

Lumen diameter (L) of the mesenteric artery was reduced in SHR, which were blunted by Ad-Sal-shRNA. In aorta, renal artery and mesenteric artery, media thickness (M) and the ratio of M to L were increased in SHR, which were prevented by Ad-Sal-shRNA (Fig. 4A,B).

### Norepinephrine and tyrosine hydroxylase

**Figure 5:**
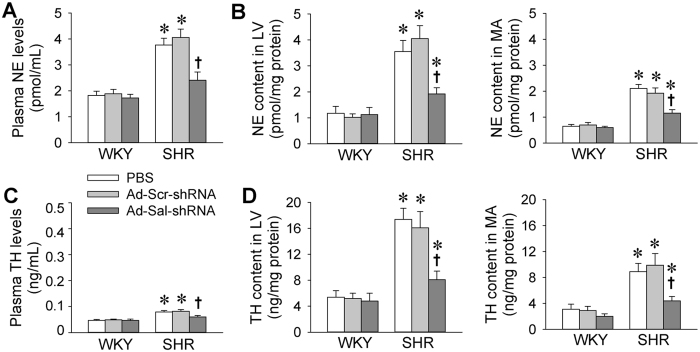
Norepinephrine and tyrosine hydroxylase in WKY and SHR. The measurements were carried out 2 weeks after intravenous injection of PBS, adenoviral vectors encoding scramble shRNA (Ad-Scr-shRNA) or salusin-β shRNA (Ad-Sal-shRNA). (**A**) Plasma norepinephrine (NE) levels. (**B**) NE contents in myocardium of left ventricle (LV) and mesenteric artery (MA). (**C**) Plasma tyrosine hydroxylase (TH) levels. (**D**) TH contents in LV and MA. Values are mean ± S.E.M. ^*^P < 0.05 vs. WKY. ^†^P < 0.05 vs. PBS or Ad-Scr-shRNA. n = 6 for each group.

Excessive sympathetic activation plays a critical role in hypertension^20^,^21^,^22^. Norepinephrine (NE) and tyrosine hydroxylase (TH) are commonly used as indirect indexes of sympathetic activity^23^,^24^. Plasma NE and TH levels were raised in SHR, which were normalized by Ad-Sal-shRNA (Fig. 5A,C). Furthermore, increased NE and HT contents in myocardium and mesenteric artery were reduced by Ad-Sal-shRNA treatment (Fig. 5B,D).

### Angiotensin system

**Figure 6:**
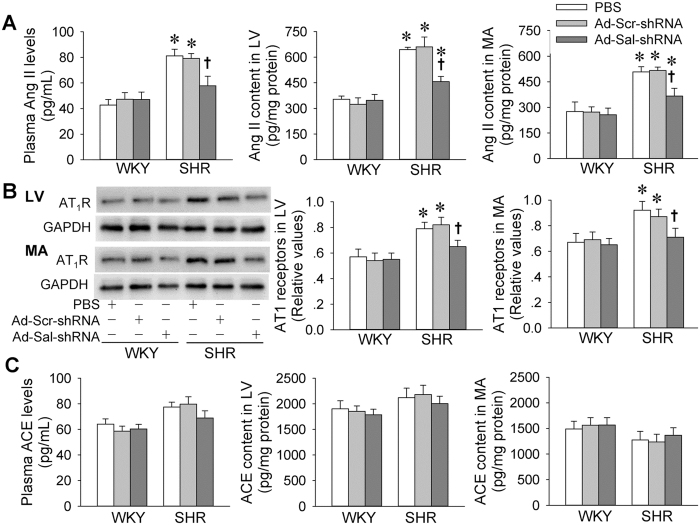
Angiotensin II, type 1 receptors of angiotensin and angiotensin converting enzyme in WKY and SHR. The measurements were carried out 2 weeks after intravenous injection of PBS, adenoviral vectors encoding scramble shRNA (Ad-Scr-shRNA) or salusin-β shRNA (Ad-Sal-shRNA). (**A**) Plasma angiotensin II (Ang II) levels and Ang II contents in myocardium of left ventricle (LV) and mesenteric artery (MA). (**B**) Type 1 receptors of angiotensin (AT_1_R) expression in LV and MA. (**C**) Plasma angiotensin converting enzyme (ACE) levels and ACE contents in LV and MA. Values are mean ± S.E.M. ^*^P < 0.05 vs. WKY. ^†^P < 0.05 vs. PBS or Ad-Scr-shRNA. n = 6 for each group.

Circulating and local angiotensin II (Ang II) is a major hormonal factor contributing to cardiovascular remodeling and hypertension^25^,^26^. Either plasma Ang II levels or local Ang II contents in myocardium and mesenteric artery were increased in SHR, which were reduced by Ad-Sal-shRNA (Fig. 6A). Type 1 receptors of angiotensin (AT_1_R) expression in myocardium and mesenteric artery were up-regulated in SHR, which were down-regulated by Ad-Sal-shRNA (Fig. 6B). However, there were no significant difference in angiotensin converting enzyme (ACE) levels in plasma, myocardium and mesenteric artery between WKY and SHR, which were not affected by administration of Ad-Sal-shRNA (Fig. 6C).

### Superoxide anions and NAD(P)H oxidase activity

**Figure 7:**
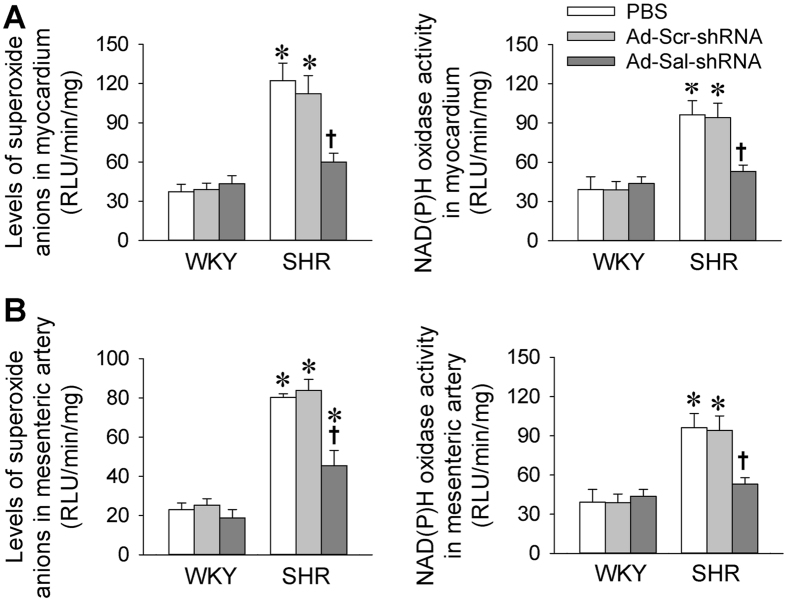
Superoxide anions levels and NAD(P)H oxidase activity in myocardium and mesenteric artery in WKY and SHR. The measurements were carried out 2 weeks after intravenous injection of PBS, adenoviral vectors encoding scramble shRNA (Ad-Scr-shRNA) or salusin-β shRNA (Ad-Sal-shRNA). (**A**) Superoxide anions levels and NAD(P)H oxidase activity in myocardium. (**B**) Superoxide anions levels and NAD(P)H oxidase activity in mesenteric artery. Values are mean ± S.E.M. ^*^P < 0.05 vs. WKY. ^†^P < 0.05 vs. PBS or Ad-Scr-shRNA. n = 6 for each group.

Reactive oxygen species (ROS) are associated with Ang II signaling and cardiovascular remodeling in hypertension^27^,^28^. Superoxide anion levels and NAD(P)H oxidase activity in myocardium were higher in SHR than those in WKY, which were normalized by Ad-Sal-shRNA (Fig. 7A). Similarly, Superoxide anion levels and NAD(P)H oxidase activity in mesenteric artery were higher in SHR than those in WKY, which were reduced by Ad-Sal-shRNA (Fig. 7B).

### Proliferation and fibrosis in VSMCs

**Figure 8:**
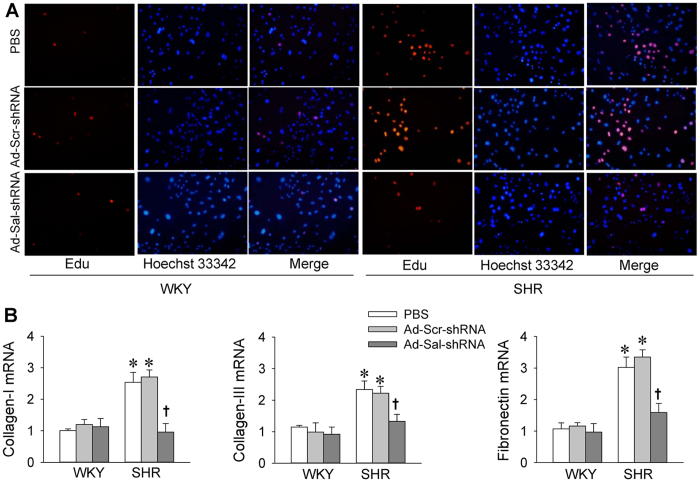
Cell proliferation and fibrosis in primary VSMCs from WKY and SHR. (**A**) VSMC proliferation was determined with EdU incorporation assay (×200). Red fluorescence (Edu) stands for cells with DNA synthesis, and blue fluorescence (Hoechst 33342) shows cell nuclei. (**B**) Fibrosis was evaluated with the collagen-I, collagen-III and fibronectin mRNA expressions. The measurements were carried out 48 h after administration of PBS, Ad-Scr-shRNA or Ad-Sal-shRNA (40 MOI). Values are mean ± S.E.M. ^*^P < 0.05 vs. WKY. ^†^P < 0.05 vs. PBS or Ad-Scr-shRNA. n = 6 for each group.

EdU incorporation assay showed that cell proliferation in vascular smooth muscle cells (VSMCs) from SHR were enhanced than those from WKY, which were inhibited by Ad-Sal-shRNA (Fig. 8A). The mRNA expressions of collagen-I, collagen-III and fibronectin, the markers of fibrosis, were increased in the VSMCs from SHR, which were down-regulated by Ad-Sal-shRNA (Fig. 8B).

## Discussion

Our previous studies have shown that intravenous administration of salusin-β dose-dependently increases blood pressure in anesthetized rats, while extra high dose of salusin-β reduces blood pressure via its bradycardia effect^10^. Moreover, salusin-β overexpression in normal rats caused persistent and severe hypertension^10^. The primary novel findings in the present study are that knockdown of salusin-β with Ad-Sal-shRNA attenuated hypertension and cardiovascular remodeling in SHR. The results indicate the importance of salusin-β in the pathogenesis of hypertension, and further support the hypertensive effect of salusin-β. Intervention of salusin-β may be a strategy for attenuating hypertension and related cardiovascular complications.

Left ventricular hypertrophy is associated with hypertension is recognized as a strong, virtually independent cardiovascular risk factor^29^. Arterial media hypertrophy in SHR is found in thoracic aorta, main renal artery and Branches III and IV of mesenteric arteries^30^. The remodeling of the large and small arteries contributes to the development and end-organ damages of hypertension^31^. Salusin-β has been found to promote human VSMC proliferation and vascular fibrosis^10^. In the present study, plasma salusin-β levels and local salusin-β contents in the myocardium of left ventricle and mesenteric artery were upregulated in SHR. Knockdown of salusin-β inhibited proliferation and fibrosis in primary VSMCs from SHR. A very inspiring result is the noticeably improved cardiovascular remodeling with Ad-Sal-shRNA treatment in SHR, as most notably evidenced by attenuated left ventricular hypertrophy, perivascular fibrosis, and vascular remodeling. These results suggest that the upregulation of salusin-β in SHR partially contributes to the cardiovascular remodeling including the proliferation and fibrosis, and down-regulation of salusin-β may be beneficial to attenuate the organ damage and cardiovascular complications of hypertension.

Ang II is associated with the genesis of arterial hypertension and cardiovascular remodeling^32^,^33^,^34^. Renin-angiotensin system intervention in hypertensive patients lowers morbidity/mortality^35^,^36^. In the present study, salusin-β gene silence normalized the increased circulating Ang II levels as well as the local Ang II contents in myocardium and mesenteric artery in SHR. Moreover, the upregulation of AT_1_ receptors in both myocardium and mesenteric artery in SHR were inhibited by knockdown of salusin-β. The inhibitory effect of salusin-β on the activation of angiotensin system may partially contributes to the attenuation of hypertension and cardiovascular remodeling. It is well known that increased oxidative stress is associated with endothelial dysfunction, inflammation, hypertrophy, apoptosis, cell migration and fibrosis in relation to vascular remodeling of hypertension^37^,^38^. Vascular ROS are derived primarily by NAD(P)H oxidases, which are prime targets for therapeutic development^39^,^40^. Previous studies in our lab have showed that salusin-β in PVN and RVLM increases sympathetic outflow and blood pressure via superoxide anions in hypertensive rats^7^,^8^. More recently, we have found that superoxide anions in VSMCs mediate salusin-β-induced foam cell formation and monocyte adhesion^16^, VSMCs migration and intimal hyperplasia after vascular injury^17^. In the present study, we found that the increased NAD(P)H oxidase activity and superoxide anion level in both myocardium and mesenteric artery of SHR were attenuated by Ad-Sal-shRNA. These results suggest that the upregulation of salusin-β in SHR partially contributes to cardiovascular remodeling via NAD(P)H oxidase-derived superoxide anion production.

Sympathetic activity is enhanced in hypertensive patients and hypertensive animal models^22^,^41^. The excessive sympathetic activity contributes to the pathogenesis of hypertension and progression of organ damage^42^. NE an TH are usually used as indirect indexes of sympathetic activity^23^,^24^. We found that plasma NE and TH levels as well as local NE and TH contents in both myocardium and mesenteric artery were raised in SHR, which were reduced by Ad-Sal-shRNA. These results suggest that knockdown of salusin-β attenuates sympathetic activation in SHR. The reduced sympathetic activity may have beneficial roles in attenuating cardiovascular remodeling and hypertension in SHR. Previous studies have showed that salusin-β is upregulated in the PVN and RVLM of the brain, and the upregulated salusin-β increases sympathetic outflow and blood pressure in 2K1C-induced renovascular hypertensive rats^7^,^8^. Blockade of salusin-β attenuates hypertension in SHR^9^. In the present study, salusin-β in the PVN and RVLM was upregulated in SHR. However, intravenous administration of Ad-Sal-shRNA had no significant role in reducing salusin-β contents in the PVN and RVLM of both WKY and SHR, suggesting that the inhibitory effect of Ad-Sal-shRNA on sympathetic activity is independent of its central effect in the present study. It is noted that Ad-Sal-shRNA attenuated hypertension and cardiovascular remodeling in SHR without significant effect on blood pressure and cardiovascular structure in WKY, although it downregulated salusin-β in both WKY and SHR. The most likely reason is that salusin-β is not involved in physiological modulation of blood pressure and cardiovascular structure, but increased salusin-β level in hypertension or some other pathological conditions contributes to hypertension and cardiovascular remodeling.

In summary, our study provides evidence that shRNA interference targeting salusin-β attenuates hypertension and myocardial and vascular remodeling in SHR. It attenuates the angiotensin and sympathetic activation as well as oxidative stress in SHR. Increased salusin-β in SHR partially contributes to the pathogenesis of hypertension and cardiovascular remodeling. Intervention of salusin-β may be a strategy against hypertension and cardiovascular remodeling.

## Materials and Methods

### Animals

Thirteen-week-old male SHR and WKY were purchased from Vital River Laboratory Animal Technology Co. Ltd (Beijing, China) and housed on a 12-h light/dark cycle in a temperature-controlled room with standard chow and tap water ad libitum. Experimental procedures were approved by the Experimental Animal Care and Use Committee of Nanjing Medical University and conformed to the Guide for the Care and Use of Laboratory Animal published by the US National Institutes of Health (NIH Publication No. 85–23, revised 1996). Acute experiments were carried out under anesthesia induced by intraperitoneal injection of urethane (800 mg/kg) and a-chloralose (40 mg/kg).

### Pretreatment with adenovirus construction salusin-β shRNA plasmids *in vivo*

Ad-Sal-shRNA and Ad-Scr-shRNA were constructed by Genomeditech Co. (Shanghai, China), which down-regulated the salusin-β expression by 75%. The sequences of salusin-β-shRNA are 5′-gatccGCCCTTCTTGGGTTGTGTATGTTCAAGAGACATACACAACCCAAGAAGGGCTTTTTTa-3′ (sense), and 5′-agcttAAAAAAGCCCTTCTTGGGTTGTGTATGTCTCTTGAACATA CACAACCCAAGAAGGGCg-3′ (antisense). The sequences of scrambled shRNA are 5′-gatccGTTCTCCGAACGTGTCACGTTTCAAGAGAACGTGACACGTTCGGAGAACTTTTTTACGCGTg-3′ (sense), 5′-aattcACGCGTAAAAAAGTTCTCCGAACGTGTCACGTTCTCTT GAAACGTGACACGTTCGGAGAACg-3′ (antisense). The efficiency of the Ad-Sal-shRNA in knockdown of salusin-β was confirmed in rats in our previous study^17^. Either WKY or SHR were randomly divided into three groups (n = 12 for each group), which were respectively subjected to injection of PBS, adenovirus expressing scrambled shRNA (Ad-Scr-shRNA) or adenovirus expressing salusinβ-shRNA (Ad-Sal-shRNA, 2 × 10^11^ plaque forming units/ml, 100 μl) via tail vein. The rats were euthanized with an overdose of pentobarbital sodium (150 mg/kg, iv) at the end of the 2nd week, and heart and blood vessels were harvested for measurements.

### Blood pressure measurement

SBP of tail artery was measured in conscious state with a noninvasive computerized tail-cuff system (NIBP, ADInstruments, Sydney, New South Wales, Australia). The rats were warmed for 10–20 min at 28 °C before the measurements in order to allow detection of tail artery pulsations and to achieve the steady pulse level. SBP was obtained by averaging 10 measurements^8^. Moreover, MAP and HR were recorded under anesthesia during acute experiments.

### Measurement of salusin-β, NE, TH and Ang II

Commercial ELISA kits were used for the measurement of salusin-β and TH (Uscn Life Science, Houston, TX, USA) as well as NE and Ang II (R&D systems, Minneapolis, MN, USA) according to the manufacturer’s descriptions. The reactions were stopped with stop solution and the final solution read at 450 nm by using a microtiter plate reader (ELX800, BioTek, Vermont, USA).

### Measurement of superoxide anion levels

Superoxide anion levels were measured using the enhanced lucigenin chemiluminescence method as we previously reported^43^,^44^. Briefly, the tissue homogenate supernatant was diluted in modified HEPES buffer. The reaction started by addition of dark-adapted lucigenin (5 μM). Light emission was measured for 10 times in 10 min with a luminometer (20/20 n, Turner, CA, USA), and the average values were calculated and expressed as relative light unit (RLU) per minute per milligram of protein.

### Measurement of NAD(P)H oxidase activity

NAD(P)H oxidase activity was measured with enhanced lucigenin chemiluminescence method as we previously reported^44^. Briefly, the tissue homogenate supernatant was diluted in modified HEPES buffer added with SOD (350 U/ml). Then, the NAD(P)H (100 μM) was added into the reaction system as a substrate for generating the superoxide anions. The reaction between superoxide anions and lucigenin started at the time of adding darkadapted lucigenin (5 μM) into the reaction system. Light emission was measured for 10 times in 10 min with a luminometer (20/20 n, Turner, CA, USA), and values were expressed as RLU per minute per milligram of protein.

### Evaluation of left ventricular hypertrophy and remodeling

Heart, left ventricle including interventricular septum and right ventricle were weighed. Then, the left ventricle tissue was fixed and sectioned (5 μm). In hematoxylin and eosin-stained sections, myocyte cross-sectional area was determined in the left ventricular lateral-mid free wall including epicardial and endocardial portions. In Masson’s trichrome-stained sections, perivascular fibrosis was evaluated in the intramuscular arteries and arterioles^45^.

### Evaluation of vascular remodeling

Descending thoracic aorta, main renal artery and the third-order branches of the mesenteric artery were dissected and washed with cold PBS for three times. Connective tissues affiliated to vessels were cleaned, and the endothelium was denuded via gentle rubbing, then the adventitia was carefully removed with forceps as previously described^46^,^47^,^48^. The arteries were embedded in paraffin, cut into 5-μm thick cross-sections, and stained with Masson’s trichrome staining. The structural changes of these arteries were observed with a light microscope. The M, L and the M/L were used as indexes of vascular remodeling^49^.

### Culture of primary VSMCs

VSMCs from rat aorta were prepared by enzymatic digestion. The VSMCs were cultured in DMEM with FBS (10%), penicillin (100 units/ml) and streptomycin (100 mg/ml) at 37 °C in a 5% CO_2_ humidified incubator. Cells in the second to sixth passages were used and cells at 80% to 90% confluence were arrested by incubating in serum-deprived DMEM for 24 hours before stimulation^17^,^50^.

### EdU incorporation assay

EdU incorporation assay was used to examine VSMC proliferation with a commercial *In Vitro* Imaging Kit (Guangzhou RiboBio, Guangzhou, China). The DNA synthesis of VSMCs was measured using a Cell-Light™ EdU Apollo^®^ 567. Red fluorescence (Edu) stands for the cells with DNA synthesis, and blue fluorescence (Hoechst 33342) shows cell nuclei.

### Real-time quantitative PCR analysis

Real-time quantitative PCR was used to examine collagen-I, collagen-III and fibronectin mRNA expression. Total RNA was isolated with Trizol reagent. The mRNA concentration in samples was measured, and 0.5 μg of total RNA was reverse transcribed to cDNA. Quantitative PCR with SYBR Premix Ex Taq TM (Takara, Otsu, Shiga, Japan) was performed in triplicates by reacting with strand-specific primers, and the average cycle thresholds were used to determine the fold-change^10^. The sequences of primers were listed in a table (Supplementary Table 1).

### Statistical analysis

Data were expressed as mean ± S.E.M. One-way or two-way ANOVA followed by post hoc Bonferroni test was used for multiple comparisons. A value of P < 0.05 was considered statistically significant.

## Additional Information

**How to cite this article:** Ren, X.-S. *et al*. Silencing salusin-β attenuates cardiovascular remodeling and hypertension in spontaneously hypertensive rats. *Sci. Rep.* **7**, 43259; doi: 10.1038/srep43259 (2017).

**Publisher's note:** Springer Nature remains neutral with regard to jurisdictional claims in published maps and institutional affiliations.

## Supplementary Material

Supplementary Information

Supplementary Dataset

## Figures and Tables

**Table 1:** Anatomic data in WKY and SHR.

Items	WKY			SHR		
	PBS	Ad-Scr- shRNA	Ad-Sal- shRNA	PBS	Ad-Scr- shRNA	Ad-Sal- shRNA
BW (g)	345 ± 8	340 ± 9	343 ± 7	309 ± 8*	311 ± 10*	317 ± 8*
HW (mg)	941 ± 19	943 ± 19	908 ± 26	1257 ± 35*	1261 ± 28*	1021 ± 31*†
LVW (mg)	748 ± 22	732 ± 24	711 ± 19	1030 ± 20*	1004 ± 27*	824 ± 20*†
HW/BW (10^−3^)	2.74 ± 0.10	2.78 ± 0.10	2.66 ± 0.11	4.06 ± 0.16*	4.07 ± 0.15*	3.24 ± 0.12*†
LVW/BW (10^−3^)	2.18 ± 0.10	2.16 ± 0.09	2.08 ± 0.08	3.34 ± 0.10*	3.24 ± 0.12*	2.61 ± 0.06*†

Measurements were carried out 2 weeks after intravenous injection of PBS, adenoviral vectors encoding scramble shRNA (Ad-Scr-shRNA) or salusin-β shRNA (Ad-Sal-shRNA). BW, body weight; HW, heart rate; LVW, left ventricular weight. Values are mean ± S.E.M. ^*^P < 0.05 vs. WKY. ^†^P < 0.05 vs. PBS or Scrambled shRNA. n = 6 for each group.
