# Supplementary material for: Silencing salusin-β attenuates cardiovascular remodeling and hypertension in spontaneously hypertensive rats
Source: Sci Rep. 2017 Feb 23;7:43259. doi: 10.1038/srep43259 (PMC5322393; doi:10.1038/srep43259)

Silencing salusin-β attenuates cardiovascular remodeling and hypertension in spontaneously hypertensive rats

Xing-Sheng Ren [1], Li Ling[1], Bing Zhou[1], Ying Han[1], Ye-Bo Zhou[1], Qi Chen[2], Yue-Hua Li[2], Yu-Ming Kang[3], Guo-Qing Zhu[1,2]*

[1]Key Laboratory of Cardiovascular Disease and Molecular Intervention, Department of Physiology, Nanjing Medical University, Nanjing, Jiangsu 211166, China; [2]Department of Pathophysiology, Nanjing Medical University, Nanjing, Jiangsu 211166, China; [3]Department of Physiology and Pathophysiology, Cardiovascular Research Center, Xi'an Jiaotong University School of Medicine, Xi'an 710061, China

Original blots for some cropped blots in Figure 6B

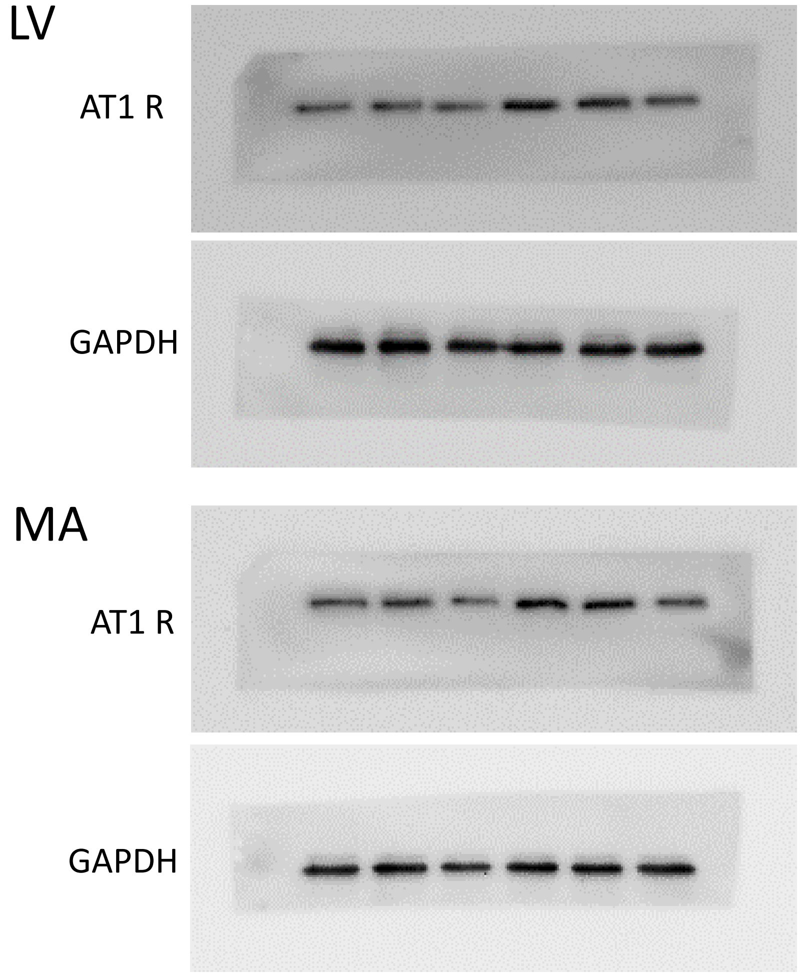

Supplement: Supplementary Information [file srep43259-s1.pdf]
