# Supplementary material for: Silencing salusin-β attenuates cardiovascular remodeling and hypertension in spontaneously hypertensive rats
Source: Sci Rep. 2017 Feb 23;7:43259. doi: 10.1038/srep43259 (PMC5322393; doi:10.1038/srep43259)
Supplement: Supplementary Dataset [file srep43259-s2.doc]

**Title Page**

**Silencing salusin-β attenuates cardiovascular remodeling and hypertension in spontaneously hypertensive rats**

Xing-Sheng Ren ^1^, Li Ling^1^, Bing Zhou^1^, Ying Han^1^, Ye-Bo Zhou^1^, Qi Chen^2^, Yue-Hua Li^2^, Yu-Ming Kang^3^, Guo-Qing Zhu^1,2^*****

^1^Key Laboratory of Cardiovascular Disease and Molecular Intervention, Department of Physiology, Nanjing Medical University, Nanjing, Jiangsu 211166, China; ^2^Department of Pathophysiology, Nanjing Medical University, Nanjing, Jiangsu 211166, China; ^3^Department of Physiology and Pathophysiology, Cardiovascular Research Center, Xi'an Jiaotong University School of Medicine, Xi'an 710061, China

**Supplemental Table**

**Supplementary Table 1** Primers for Real-time quantitative PCR analysis in rat VSMCs.

|  | Primer | Sequence | Accession number |
| --- | --- | --- | --- |
| Collagen I | Forward | 5'-GAGCCTAACCATCTGGCATCT-3' | NM-053304.1 |
|  | Reverse | 5'-AGAACGAGGTAGTCTTTCAGCAAC-3' |  |
| Collagen III | Forward | 5'-AGATGCTGGTGCTGAGAAG-3' | NM-032085.1 |
|  | Reverse | 5'-TGGAAAGAAGTCTGAGGAAGG-3' |  |
| Fibronectin | Forward | 5'-GTGAAGAACGAGGAGGATGTG-3' | XM-006245159.1 |
| GAPDH | Reverse  Forward  Reverse | 5'-GTGATGGCGGATGATGTAGC-3'  5'-GGAAAGCTGTGGCGTGAT-3'  5'-AAGGTGGAAGAATGGGAGTT-3' | NM-017008.4 |
